# Supplementary material for: A Novel Route to High-Quality Graphene Quantum Dots by Hydrogen-Assisted Pyrolysis of Silicon Carbide
Source: Nanomaterials (Basel). 2020 Feb 6;10(2):277. doi: 10.3390/nano10020277 (PMC7075118; doi:10.3390/nano10020277)
Supplement: Supplementary file 1 [file nanomaterials-10-00277-s001.pdf]

Supplementary Information

A Novel Route to High-Quality Graphene Quantum Dots by Hydrogen-Assisted Pyrolysis of Silicon Carbide

Na Eun Lee [1], *Sang Yoon Lee* [1], *Hyung San Lim* [1], *Sung Ho Yoo* [1], *Sung Oh Cho* [1,*]

[a] Department of Nuclear and Quantum Engineering, Korea Advanced Institute of Science and Technology (KAIST), Daejeon 34141, Republic of Korea

* Correspondence: socho@kaist.ac.kr; Tel.: +82-(0)42-350-3823 Fax: +82-(0)42-350-3810

KEYWORDS: graphene quantum dots; silicon carbide; hydrogen-assisted pyrolysis; high-quality

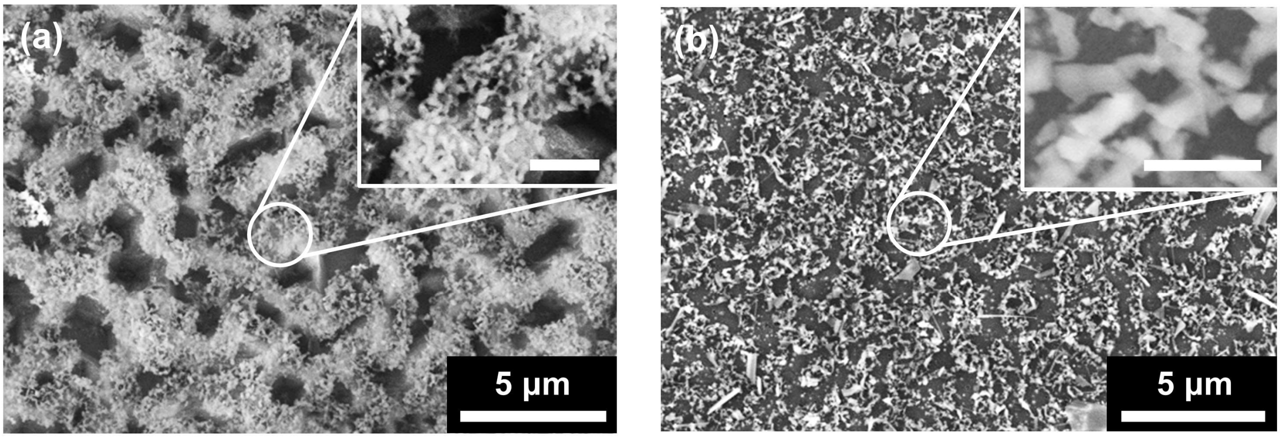

Figure S1. FE-SEM image of GQDs on SiC plate after annealed at 1500 °C with various operating hydrogen etching gas pressure. The operating pressure was **(**a**)** 120 mTorr (inset scale bar is 1 μm) and **(**b**)** 160 mTorr (inset scale bar is 1 μm).

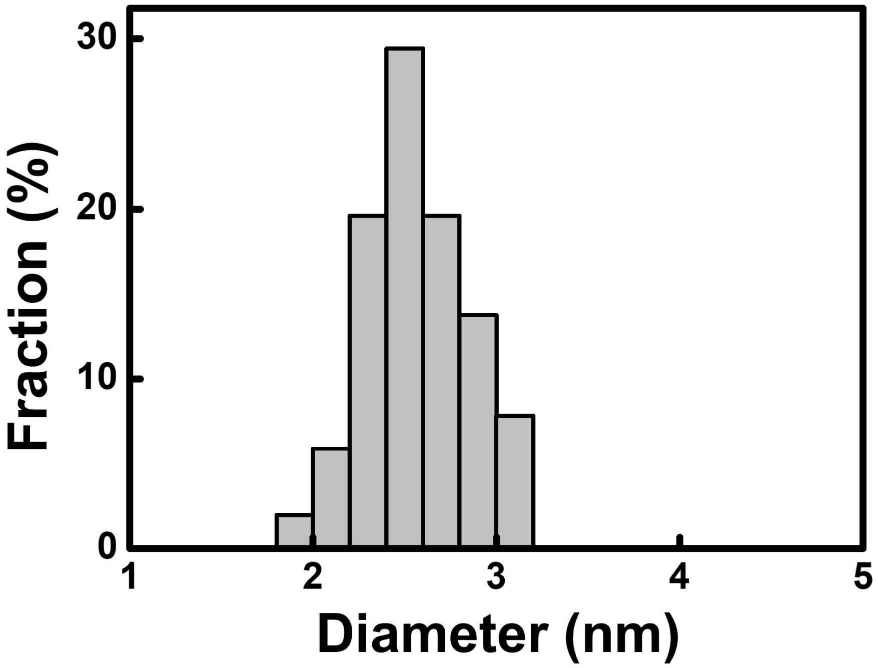

Figure S2. Size distribution of fabricated GQDs.

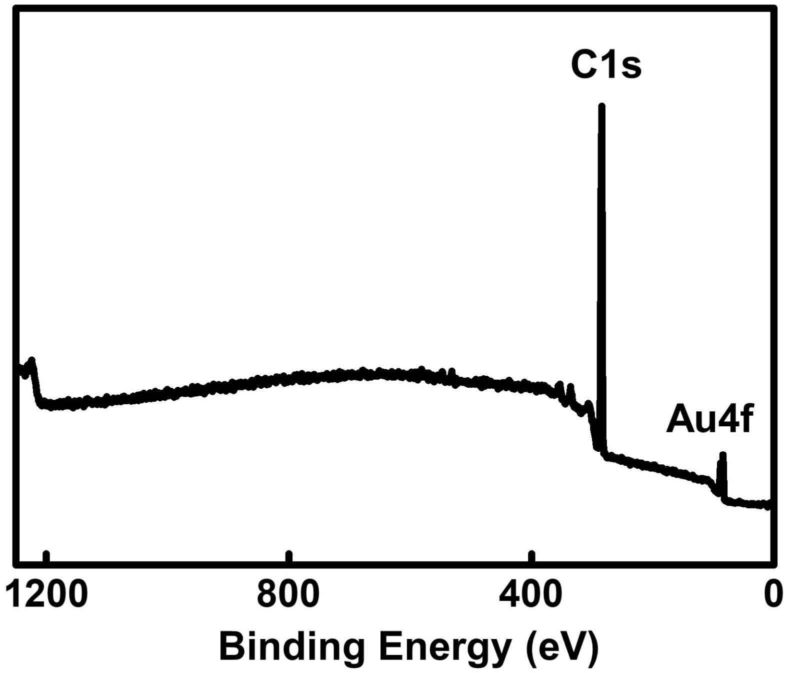

Figure S3. X-ray photoelectron spectroscopy (XPS) survey spectrum of the fabricated high-quality GQDs.
